# Preparing for Disease X: Ensuring Vaccine Equity for Pregnant Women in Future Pandemics

**DOI:** 10.3389/fmed.2022.893292

**Published:** 2022-05-30

**Authors:** Flor M. Munoz, Clare L. Cutland, Christine E. Jones, Beate Kampmann, Asma Khalil, Esperança Sevene, Andy Stergachis, Geeta K. Swamy, Gerald Voss, Ajoke Sobanjo-ter Meulen

**Affiliations:** ^1^Division of Infectious Diseases, Molecular Virology and Microbiology, Department of Pediatrics, Baylor College of Medicine, Houston, TX, United States; ^2^African Leadership in Vaccinology Expertise, Faculty of Health Sciences, University of the Witwatersrand, Johannesburg, South Africa; ^3^Faculty of Medicine and Institute for Life Sciences, National Institute for Health and Cancer Research (NIHR) Southampton Biomedical Research Centre, University of Southampton, NIHR Southampton Clinical Research Facility, University Hospital Southampton National Health Service (NHS) Foundation Trust, Southampton, United Kingdom; ^4^The Vaccine Centre, London School of Hygiene and Tropical Medicine, London, United Kingdom; ^5^Fetal Medicine Unit, Department of Obstetrics and Gynaecology, St George's University Hospitals NHS Foundation Trust, London, United Kingdom; ^6^Molecular and Clinical Sciences Research Institute, St George's University of London, London, United Kingdom; ^7^Department of Physiological Sciences, Clinical Pharmacology, Faculty of Medicine, Eduardo Mondlane University, Maputo, Mozambique; ^8^Manhiça Health Research Centre, Maputo, Mozambique; ^9^School of Public Health, University of Washington School of Pharmacy, Seattle, WA, United States; ^10^Duke University School of Medicine, Durham, NC, United States; ^11^Coalition for Epidemic Preparedness Innovations, Oslo, Norway; ^12^Bill & Melinda Gates Foundation, Seattle, WA, United States

**Keywords:** pregnant women, disease X, vaccine equity, preparedness, collaboration, coordination

## Abstract

Disease X represents a yet unknown human pathogen which has potential to cause a serious international epidemic or pandemic. The COVID-19 pandemic has illustrated that despite being at increased risk of severe disease compared with the general population, pregnant women were left behind in the development and implementation of vaccination, resulting in conflicting communications and changing guidance about vaccine receipt in pregnancy. Based on the COVID-19 experience, the COVAX Maternal Immunization Working Group have identified three key factors and five broad focus topics for consideration when proactively planning for a disease X pandemic, including 10 criteria for evaluating pandemic vaccines for potential use in pregnant women. Prior to any disease X pandemic, collaboration and coordination are needed to close the pregnancy data gap which is currently a barrier to gender equity in health innovation, which will aid in allowing timely access to life-saving interventions including vaccines for pregnant women and their infants.

## Introduction

In the past decades, substantial advances have been made in the field of maternal immunization, which is well recognized as an effective public health measure to reduce the burden of certain maternal and infant infectious diseases (1). Although vaccines specifically designed for administration during pregnancy (e.g., for Group B streptococcus and respiratory syncytial virus) are now entering late-stage clinical development, the inclusion of pregnant women in clinical trials of vaccines not specifically targeted for use during pregnancy remains an under-prioritized area, as experienced in the context of SARS-CoV-2 vaccine development.

Globally, an estimated 180 million pregnancies occur annually, with the vast majority in low- and middle-income countries (LMICs) (2). The COVID-19 pandemic has reinforced the fact that pregnant women are an at-risk population for emerging infectious diseases. Therefore, in a pandemic situation, pregnant women need to be able to access potential life-saving interventions concurrently with the rest of the population. Any delay in access, for example due to waiting for comprehensive long-term safety data, places these women at risk of preventable maternal morbidity or mortality, as well as adverse pregnancy outcomes.

The lack of timely consideration of the potential needs of pregnant women was particularly stark during the current COVID-19 pandemic. Pregnant women, especially those >35 years of age or with comorbidities such as obesity or hypertension were shown to be at an increased risk of severe COVID-19 and its complications, including higher odds of intensive care unit (ICU) admission, invasive ventilation, preterm delivery, stillbirth, or maternal death (3–7). This, together with the fact that women of child-bearing age made up a significant portion of the global frontline healthcare workforce, or were often employed in other occupations at high-risk of SARS-CoV-2 exposure, meant that this vulnerable group should have been given high priority for receiving COVID-19 vaccines (8). However, while pregnant women are being increasingly recognized as a critical population for inclusion in vaccine research, they were excluded from efficacy/pivotal clinical trials for all COVID-19 candidate vaccines, consistent with standard clinical development practices and the pressures to achieve regulatory approval rapidly. As a consequence, at the time of the first COVID-19 vaccine emergency use approvals, data in pregnant women were lacking and developmental and reproductive toxicity (DART) studies were still ongoing.

Addressing the known heightened risk for severe COVID-19 disease outcomes in pregnancy, several professional societies and other vaccine recommending bodies issued permissive recommendations allowing pregnant women access to vaccination based on the clinical safety profile of the vaccine in non-pregnant adults. These permissive recommendations in many countries, mostly high-income countries (HICs), allowed many pregnant women to gain access to COVID-19 vaccination in consultation with their healthcare providers. However, despite the increased risk of severe COVID-19 in pregnant women, the lack of clinical vaccine safety data in this population from a broader range of COVID-19 vaccines, together with lack of clear regulatory consensus and conflicting messaging globally has hampered access to vaccines for many women globally, particularly those in LMICs.

The implementation of linked pregnancy and vaccine surveillance systems to rapidly collect safety information during the post-Emergency Use Authorization (EUA) approval period has now provided vital safety data (9, 10), but this remains restricted to vaccines approved in HICs where these systems exist, and does not include many of the vaccines which are the only options currently available to LMICs.

Recognizing the importance of addressing the needs of pregnant women during the COVID-19 pandemic, the COVAX COVID-19 Maternal Immunization Working Group (MIWG)—a cross-disciplinary team of experts from clinical medicine, regulatory affairs, ethics, clinical research, pharmacovigilance, and vaccine safety—developed criteria to assess the suitability of pandemic vaccine candidates for roll-out to pregnant women. In addition, the COVAX MIWG identified a number of unmet needs in data availability, study designs and implementation, communication, and regulatory considerations to facilitate COVID-19 vaccine access for pregnant women worldwide. Here we describe these criteria and unmet needs which will be of value to proactively plan for future Disease X pandemics.

## Evaluating Potential Vaccine Candidates for Use in Pregnant Women

Based on the COVID-19 experience, we identified ten major criteria to optimize the evaluation of pandemic vaccines for use in pregnant women (Table 1). These criteria were established based on expert discussions, evaluation of ongoing vaccine safety data, and mapping of vaccine candidate characteristics. At a minimum, data from DART studies (either for the specific vaccine or the same platform) and from clinical trials in non-pregnant adults demonstrating vaccine safety and immunogenicity should be available prior to enrolment of pregnant women in clinical trials of pandemic vaccine candidates. Trials of vaccine candidates in non-pregnant adults provide an important source of safety and immunogenicity data that may be extrapolated to pregnant women in a pandemic situation, together with any data from inadvertent exposures in clinical trial participants who became pregnant during the study. Maternal and infant outcomes from pregnant women inadvertently exposed or enrolled in vaccine trials should be monitored by clinical and laboratory measures to assess for potential increased reactogenicity and adverse events compared with the general population (11, 12). While efficacy trials do not necessarily have to include pregnant women, their exclusion prevents the collection of valuable data on both safety and efficacy during pregnancy, and should be considered if there are no factors which indicate against their inclusion. Post-EUA or post-licensure studies and pregnancy registries should be utilized for continuing assessments of maternal and infant outcomes, and a minimum follow-up period of 6 months for infant safety should be applied, with preference for at least 12 months of follow-up.

**Table 1:** Lessons learned from the COVID-19 pandemic: pro-active planning considerations for Disease X.

**Domain**	**Needs**
Evaluation of potential pandemic vaccines for roll-out to pregnant women	1. Results of DART studies, with advice to developers to plan for expedited conduct, analysis, and release of DART data 2. Safety data available from use of the same vaccine platform^*^ in non-pregnant populations 3. Safety data available from use of the same vaccine candidate in non-pregnant populations 4. Prior data on the antigen and delivery system/platform (subunit, non-replicating vector, vectored, mRNA, DNA, etc.) in pregnant populations 5. Safety data on adjuvant-antigen pair testing in non-pregnant adults, along with data from DART studies for the adjuvanted construct, when applicable 6. Safety data from clinical trials in pregnant and lactating women of vaccines utilizing the same adjuvant as the candidate vaccine (as appropriate) 7. Safety data from participants in clinical trials who have become pregnant whilst in the trial or data from inadvertent exposures during 8. Available local and systemic reactogenicity profile of the candidate vaccine in non-pregnant population as an indication of possible local and systemic reactogenicity in pregnant women, with particular interest in fever after vaccination 9. Data from post-licensure studies and vaccine registries when planning post-licensure studies in pregnant women 10. Plan for safety follow up in place: minimum through delivery for the mother, and minimum 6-month (preferred: 12-month) post-delivery follow-up for infant safety evaluation
Data needs	Collection of background rates of maternal and infant outcomes, particularly in LMICs Collection of disease burden data for Disease X across different epidemiological settings, once Disease X identified Earlier collection of DART data in clinical development plans
Regulatory considerations	Harmonized guidance in place for consideration of pregnant women during vaccine development Standardized protocols and frameworks for vaccine effectiveness studies which can be utilized globally Proactive presumption of inclusion of pregnant women in clinical development rather than exclusion
Pharmacovigilance	Development of systems which can be utilized to collect data during a pandemic Leverage existing systems for passive collection of data (e.g., obstetrics monitoring systems) Funding, development, and utilization of pregnancy registries for vaccine pharmacovigilance, particularly in LMICs
Communication and overcoming vaccine hesitancy	Proactive recruitment of professional vaccination champions who live in the regions of people being vaccinated Data and information about vaccination during pregnancy and lactation to be accessible to the general public, healthcare providers and policy makers, in addition to scientific communities Proactive preparation of clear messages of the benefits of vaccines and medications during pregnancy Effective utilization of social media platforms Positivity of messaging (rather than e.g., lack of concerning safety signals)

*DART, developmental and reproductive toxicology; LMIC, low and middle-income country*.

* *Same vaccine platform refers to the same or highly similar construct previously used to create a vaccine against another pathogen*.

## Factors Enabling Vaccination of Pregnant Women During a Pandemic

**Figure 1:**
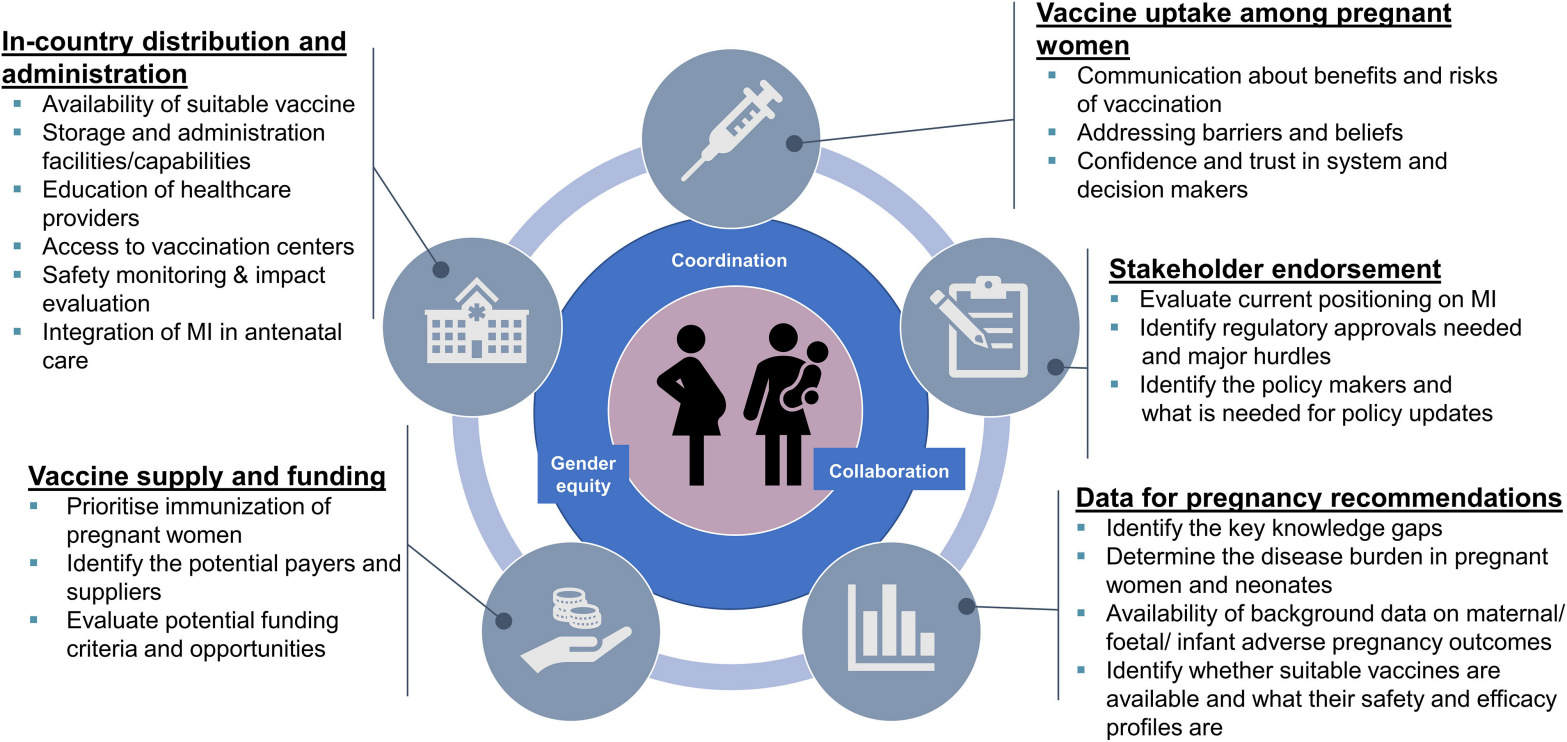
Enabling factors for pandemic vaccination of pregnant women.

Three key factors and five broad focus topics were identified as critical enabling factors for vaccination of pregnant women during a pandemic situation (Figure 1). Gender equity, coordination, and collaboration were identified as cross-theme key factors which should be considered for each of the five focus topics. Gender equity is an often-overlooked factor that should be an important consideration at all levels going forward. At a scientific level, gender impacts are not routinely assessed in randomized clinical trials or observational studies, therefore data on the effects of a drug or biologic are generalized to the entire population, which may create a data gap regarding the effectiveness and safety in women and hence pregnant women. This lack of data may create a barrier to access to potentially life-saving interventions for these women. At a social level, gender inequity still significantly impacts access to healthcare, particularly in LMICs (13). For example, robust and timely safety and effectiveness data in pregnant women are needed to enable vaccine policy decisions and access to vaccines for pregnant women, and should be an important consideration for drug development going forward. Coordination between vaccine- and maternal and neonatal health experts and stakeholders from vaccine development through to implementation are vital to ensure timely vaccine access and rapid uptake and coverage in pregnant women. The third factor, collaboration between stakeholders also plays an important role in ensuring timely access to vaccines, including collection and sharing of data between vaccine developers and regulatory bodies, ensuring vaccine availability, and communication of clear messages to encourage uptake of vaccines by pregnant women. An international body such as the WHO would be the most appropriate umbrella organization for ensuring effective worldwide coordination and collaboration during a Disease X pandemic.

Across these three factors, five focus topics have been identified (Figure 1). The first focus topic emphasizes the need for data on the disease burden and outcomes in pregnant women and neonates and the suitability of current/upcoming vaccines to support recommendations. A second topic is stakeholder endorsement including clear guidance and positioning on maternal immunization from recommending bodies and professional societies, as well as timely policy updates as new data emerge. Thirdly, considerations about enabling vaccine supply include prioritization in vaccination tiers and identifying the potential financers and funding criteria for vaccination of pregnant women. The fourth broad topic identified was enabling of vaccine distribution and administration, both internationally and within countries. This includes ensuring availability of a vaccine from a suitable platform for administration during pregnancy or lactation, education of healthcare providers, ensuring access to vaccination sites, and ensuring safety monitoring and evaluation systems are in place for timely identification of any potential safety signals. Finally, maximizing vaccine uptake among pregnant women includes communication measures (e.g., social media channels) to specifically communicate benefits of vaccination vs. disease risk, addressing potential barriers to vaccination, and gaining trust and confidence in both the system and decision makers for this specific group of vaccine recipients.

## Lessons Learned From COVID-19: How Can These Benefit the Disease X Pandemic Response?

The COVID-19 experience has highlighted a number of areas where proactive preparation for a future Disease X pandemic could greatly improve rapid access to pandemic vaccines for pregnant women (Table 1). Data on background rates of maternal and fetal outcomes are lacking for many LMICs, and such data collection should be prioritized so that any impacts of disease or vaccination on these outcomes can be accurately evaluated. Existing pregnancy registries and safety surveillance systems can play a significant role in collecting these data and can therefore be utilized to estimate disease burden and vaccine effectiveness during a pandemic.

An important lesson learnt from the current pandemic is the critical need for harmonized guidance and data systems to be in place prior to initiating clinical vaccine development. Both global collaboration of important stakeholders as well as effective coordination between regulatory bodies, manufacturers, logistics, governmental leaders, professional societies, and educators is required to ensure that the appropriate data can be collected across vulnerable populations from the outset. To enable consistency globally, this would ideally be overseen by the WHO acting as a worldwide coordination and knowledge hub, with implementation performed at country-level. To enable timely vaccine access for pregnant women, generation of DART data on new vaccine platforms should be initiated as early as possible, before or in parallel with clinical trials, so that they are available as soon as safety, immunogenicity, and efficacy in non-pregnant populations has been established. Regulatory and policy requirements should be identified as early as possible, also for specific populations such as pregnant women to enable timely and robust decision-making. Furthermore, funding should be made available to address knowledge gaps in available vaccine platforms outside of a pandemic situation where possible, to consolidate the knowledge base well ahead of the next pandemic.

Another key lesson learned from the COVID-19 experience is the importance of effective communication of vaccine safety in mitigating potential hesitancy. Coincidental events can be very damaging to public confidence in a vaccine, particularly in pregnant women who are often highly concerned about vaccine safety both for themselves and for their infant (14, 15). Navigating perceived risk in a climate of mass social media, together with conflicting and changing national and international vaccination recommendations and hesitancy from healthcare providers compounds this issue. Inclusion of pregnant women early in future vaccine development, together with effective communication with the public and healthcare providers are a prerequisite to providing robust information on the risk-benefit profile of the vaccine vs. the disease and can ultimately increase public confidence in both the vaccine and the advice about its usage. Added to this, safety data collected via pregnancy registries under EUAs [e.g., V-safe (16)], together with specific safety epidemiological studies in pregnancy and frequent reviews of safety data [e.g., systematic reviews (17)] are vital for identification of any potential safety signals and to help increase confidence in the safety of the vaccines in these populations.

The introduction of a pandemic vaccine requires risk communication planning in advance of product launch, in order to be prepared to address safety issues as and when they may arise, as well as to mitigate vaccine hesitancy. Lack of public trust is often an overlooked factor leading to vaccine hesitancy, thus schemes which pro-actively involve pregnant women in science and innovation can help gain trust in the system and medical developments, as well as improving health.

As experienced during the COVID-19 pandemic, vaccine roll-out in HICs can occur rapidly, however access to vaccines in LMICs can be very limited, owing to greater buying power of HICs and vaccine nationalism (18, 19). Following the early approval of mRNA COVID-19 vaccines and a favorable safety profile in non-pregnant persons, HICs have made these vaccine widely available to pregnant women which has enabled safety and effectiveness data collection in tens of thousands of pregnant women. In contrast to HICs, the majority of the 90 million pregnant women in sub Saharan Africa and South East Asia still do not have access to COVID-19 vaccines, either due to lack of access or restrictive vaccination policies. To mitigate these potential inequalities in future pandemics, it is paramount that access to vaccines should be agreed early in the planning stages jointly with manufacturers, thus avoiding contractual agreements which restrict equitable global supply. Further, vaccine developers can aid in reducing inequality by performing clinical trials in LMICs, which provides vital safety and efficacy data in these settings and aids in approval and prioritization of vaccination of pregnant women by national governments.

In conclusion, pro-active planning, networking, funding support, advocacy from trusted recommending organizations, confidence and education on how and when to include pregnant women in pandemic vaccine development are paramount to ensure timely data collection and access to vaccines. Prior to a future Disease X pandemic, pro-active identification and consideration of other factors affecting vaccine delivery to pregnant women should already be in place to allow a harmonized and effective rollout of vaccines to pregnant women globally. Finally, the COVID-19 experience has taught us that we need to close the pregnancy-related data gap that is currently a barrier to gender equity in health innovation. This will enable timely access to life-saving interventions for pregnant women, reducing preventable deaths in pregnant women and their infants globally.

## Data Availability Statement

The original contributions presented in the study are included in the article/supplementary material, further inquiries can be directed to the corresponding author/s.

## Author Contributions

All authors contributed to the expert discussions resulting in the findings in this manuscript, contributed to the development of the manuscript, and approved the final version for submission.

## Funding

Funding for this study was provided by the Coalition for Epidemic Preparedness Innovations (CEPI). Editorial assistance in the preparation of this manuscript was provided by Dr. Jenny Engelmoer (Sula Communications), funded by the Bill and Melinda Gates Foundation. BK and CJ are supported by the IMmunising PRegnant women and INfants neTwork (IMPRINT) funded by the GCRF Networks in Vaccines Research and Development which was co-funded by the MRC and BBSRC.

## Conflict of Interest

FM—member DSMB Pfizer, Moderna, Meissa Vaccines, Virometix, US National Institutes of Health (NIH); Research support Pfizer, Gilead, US Centers for Disease Control and Prevention (CDC), US National Institutes of Health (NIH) and Infectious Diseases Clinical Research Center (IDCRC); Member Immunization Expert Group at the American College of Obstetrics and Gynecology (ACOG), Member Committee of Infectious Diseases (COID) of the American Academy of Pediatrics (AAP), Member Executive Committee of the Society of Infectious Diseases (SOID) of the AAP. CC—scientific advisor for Pfizer's maternal immunization work. Research and training support from BMGF, Sanofi. CJ—investigator for clinical trials sponsored by Pfizer and Medicago; participation of data safety monitoring board or advisory board for MSD, Pfizer, and Sanofi. BK—investigator for clinical trials sponsored by Pfizer; member of ad hoc Scientific Advisory Committees for GSK, Pfizer, Johnson & Johnson and member of Independent Data Monitoring Committee J&J; Director of IMPRINT https://www.imprint-network.co.uk. AS—research support from the Bill & Melinda Gates Foundation. GS—chair of Independent Data Monitoring Committees for GlaxoSmithKline (RSV vaccine) and Pfizer (Group B Strep vaccine), Member DSMB National Cancer Institute (HPV vaccine), Member Immunization Expert Group at the American College of Obstetrics and Gynecology. Investigator for clinical trials sponsored by Novavax, GlaxoSmithKline, Regeneron, Pfizer, NIH, and US Centers for Disease Control & Prevention. The remaining authors declare that the research was conducted in the absence of any commercial or financial relationships that could be construed as a potential conflict of interest.

## Publisher's Note

All claims expressed in this article are solely those of the authors and do not necessarily represent those of their affiliated organizations, or those of the publisher, the editors and the reviewers. Any product that may be evaluated in this article, or claim that may be made by its manufacturer, is not guaranteed or endorsed by the publisher.
